# An Introduction to Nonlinear Integrated Photonics Devices: Nonlinear Effects and Materials

**DOI:** 10.3390/mi14030604

**Published:** 2023-03-06

**Authors:** Luigi Sirleto, Giancarlo C. Righini

**Affiliations:** 1 National Research Council (CNR), Institute of Applied Sciences and Intelligent Systems (ISASI), Via Pietro Castellino 111, 80131 Napoli, Italy; 2 National Research Council (CNR), Institute of Applied Physics (IFAC) “Nello Carrara”, Via Madonna del Piano 10, 50019 Sesto Fiorentino, Florence, Italy

**Keywords:** photonics devices, nonlinear photonics, integrated photonics, photonic structures, optical materials, all-optical signal processing, all-optical computing, nonlinear optical sources

## Abstract

The combination of integrated optics technologies with nonlinear photonics, which has led to the growth of nonlinear integrated photonics, has also opened the way to groundbreaking new devices and applications. Here we introduce the main physical processes involved in nonlinear photonics applications, and we discuss the fundaments of this research area, starting from traditional second-order and third-order phenomena and going to ultrafast phenomena. The applications, on the other hand, have been made possible by the availability of suitable materials, with high nonlinear coefficients, and/or by the design of guided-wave structures, which can enhance the material’s nonlinear properties. A summary of the most common nonlinear materials is presented, together with a discussion of the innovative ones. The discussion of fabrication processes and integration platforms is the subject of a companion article, also submitted for publication in this journal. There, several examples of nonlinear photonic integrated devices to be employed in optical communications, all-optical signal processing and computing, or quantum optics are shown, too. We aimed at offering a broad overview, even if, certainly, not exhaustive. We hope that the overall work could provide guidance for those who are newcomers to this field and some hints to the interested researchers for a more detailed investigation of the present and future development of this hot and rapidly growing field.

## 1. Introduction

Photonics has often been defined as the key technology of the 21st century. The term “photonics” has certainly been coined in the 20th century, even if there is some incertitude on the precise creation date of this word. Some ambiguity also remains about its frontiers and the differences with respect to optoelectronics and electro-optics. One of the claims is that the first appearance of the word was in 1952 [1]. However, many authors consider the French scientist Pierre Agrain as the “father” of photonics, in 1967, but likely there was an almost simultaneous invention of the word by a group of French physicists working in lasers and fiber optics and by a Dutch group of high-speed photography specialists. A very interesting analysis of the use of the term photonics, embracing history, philosophy, and sociology of science, was published some years ago [2].

The word photonics, however, started to be broadly used only in the 1980s, when the operators of telecommunications networks switched from electrical to fiber-optic data transmission. Optical fiber systems are typically composed of discrete elements, such as lasers, modulators, and detectors often packaged in a rack scale module. In 1969, inspired by the rapid development of integrated electronics, the concept of integrated optical circuits (IOCs) was proposed by researchers at Bell Labs [3]. Some twenty years later, the growth in complexity of the optical architectures led to the introduction of the term photonic integrated circuits (PICs), and in 1991 a paper, again by researchers at AT&T Bell Labs, presented an early review of InP-based PICs [4]. Then, it took more than a decade to have the announcement of a breakthrough in photonic integration with the industry’s first large-scale PIC: a 2005 article described a 100 Gb/s dense wavelength division multiplexed (DWDM) transmitter and receiver PIC, fabricated through the integration of over 50 discrete functions onto a single monolithic InP chip [5].

In most cases, the response of a material to an applied optical field is linear (i.e., the strength of the response is proportional to the strength of the optical field), but all the way back in the second half of the 19th century, John Kerr, in Glasgow, observed effects that were proportional to the square of the applied field. These effects, which can be associated with the birth of nonlinear optics, described how an isotropic transparent substance becomes birefringent when it is placed in an electric field: two papers were published in 1875, the first related to a solid dielectric [6] and the second to liquids [7]. Another pioneering work, predicting quantum two-photon phenomena, was performed at the end of the 1920s by Maria Goeppert-Mayer (a German physicist who moved to the United States in 1930) [8]. The field of nonlinear optics, however, became much more important and application-effective after the discovery of the laser and the wide availability of intense light beams. The center of the research moved from Europe to the United States, where around 1960, the seminal studies by Peter Franken [9] and Nicolaas Bloembergen (a Dutch physicist who had moved to the United States in 1945) [10,11] actually opened the route to the amazing development of science and technology related to nonlinear optical phenomena. The first nonlinear optics experiment in waveguides, showing second-harmonic generation (SHG) in GaAs waveguides, was reported in 1971 by Anderson and Boyd, from the North American Rockwell Science Center [12].

Since the efficiency of nonlinear interaction depends upon the interacting beam intensities (power/area) and is also proportional (either linearly or quadratically) to the interaction distance, it soon became clear that optical waveguides offer important advantages. First, the confinement of light intensity within an area comparable to the wavelength of light leads to an enhanced field strength and high power density; second, the diffractionless propagation in one or two dimensions results in interaction lengths over a distance (at least of the order of centimeters, if not longer) much longer than the one obtained within a bulk material. Definitely, waveguide geometries offer the best prospects for optimizing the efficiency of nonlinear devices. Therefore, the subject of nonlinear integrated optics (NLIO) and, more broadly, nonlinear integrated photonics (NLIP) has greatly expanded due to the development of novel light sources, advanced materials, and very effective guiding structures [13,14,15,16,17,18,19,20,21,22].

The materials of interest for realizing nonlinear optical devices are typically in bulk form with interaction lengths in the millimeter range to obtain efficient buildup of the nonlinear signal of interest. In nonlinear integrated photonics, nonlinear optical effects must be exploited within photonics structures with dimensions comparable to or much less than the incident light wavelength. In this scenario, nonlinear properties of optical materials have become of paramount importance, with a dual role: on one side as sources of detrimental effects (such as limiting effects in high-power fiber lasers and amplifiers) and on the other side as essential elements to achieve a number of functions (such as light generation and modulation). Ideally, the best material should have large nonlinear susceptibilities of second and third order, ultrafast response time, and low linear and nonlinear losses (e.g., due to two-photon absorption and free-carrier absorption) within the desired wavelength ranges and the availability of mature manufacturing processes. The latter are crucial for practical applications, requiring multiple active and passive components.

The paper is organized as follows. In the next section, the fundamental aspects of the most common parametric NLO effects, that is, second-order nonlinear processes (second-harmonic generation (SHG), sum/difference frequency generation (SFG/DFG), and optical parametric amplification (OPA)) and third-order nonlinear processes (third-harmonic generation (THG), four-wave mixing (FWM), Kerr effect, SRS, and SBS) are described. As optical materials with favorable properties are the foundation to promote integrated photonic devices with large bandwidth, high efficiency, and flexibility in high-volume chip-scale fabrication, Section 3 is dedicated to optical materials of interest for nonlinear photonics.

## 2. Fundamental Phenomena

The first described nonlinear phenomenon was the electro-optical Pockels effect in 1906 by the German physicist Friedrich Pockels. In this effect, occurring only in non-centrosymmetric materials, the refractive index of a medium is modified in proportion to the applied electric field strength, which can be applied to the medium either longitudinally or transversely to the light beam. Transverse voltage requirements can be reduced by lengthening the crystal. The electro-optical Pockels effect is used in many applications, for example, EO modulation, high-speed optical shutters, electro-optical detection, and electro-optical switching. The most important material for these applications is lithium niobate (LiNbO_3_), which has been used for decades in long-haul telecommunication [23]. 

Unlike linear effects, nonlinear phenomena are subject to symmetry constraints. Second-order nonlinear optical interaction can occur only in noncentrosymmetric materials (they do not display inversion symmetry); this means that in centrosymmetric materials, the nonlinear optical susceptibility $\chi^{\left(2\right)}$ is zero, whereas third-order nonlinear interaction can occur in all materials. A fundamental feature of nonlinear processes is that they can give rise to the exchange of energy among electromagnetic fields with different frequencies. In this regards, nonlinear processes can be divided in two classes [24,25,26]:

In parametric processes, which include both second-order (second-harmonic generation (SHG) and sum or difference frequency generation (SFG and DFG)) and third-order (third-harmonic generation (THG) and four-wave mixing (FWM)) nonlinear phenomena, energy conservation and momentum conservation (i.e., the phase matching) must be satisfied. The fundamental point is that the material’s energy does not take part in the processes, and the energy transfer can occur only among waves. Parametric processes are related to non- or near-resonant interactions, where the initial and final quantum states are the same; this means that the transitional electron populations on the energy levels return to the initial condition, that is, there is no real absorption of photons. In a parametric process, the population can be removed from the ground state only for those brief intervals of time when it resides in a virtual level. Therefore, their lifetimes are extremely short (less than a femtosecond). They are always described by a real susceptibility.

To enhance the efficiency of parametric process, the condition of phase matching must be satisfied. This means that the phases of the incident and resultant waves must be in synchronism. Generally, phase matching becomes a major challenge and a constraint that limits the practical applications of the parametric process. On the other hand, because the phase matching condition can be satisfied by only one of the frequency components of the nonlinear polarization, it can work also as an effective method to select a single nonlinear interaction among many possible interactions.

In non-parametric processes, the energy of the whole system, including the material, is conservative, and the energy transfers between waves and materials exist simultaneously. As a result, part of the wave energy transfers to other energy types in the material, such as the optical phonon in the case of stimulated Raman scattering (SRS), the acoustic phonon in the case of stimulated Brillouin scattering (SBS), and the electron level in two-photon absorption (TPA). Non-parametric processes are related to resonant interactions, involving real energy levels with different initial and final quantum states, whose population are changed. In these processes, there is energy transfer from the photons to the host medium with a relative longer lifetime. We note that non-parametric processes are described by a complex susceptibility.

### 2.1. Second-Order Phenomena

In these nonlinear effects, the nonlinear polarization (P^(2)^), which is proportional to the product of two optical fields through the second-order nonlinear susceptibility, radiates electric fields at the nonlinear frequency, growing linearly with distance propagation. The second-order nonlinearities are of great importance for nonlinear photonic applications, providing valuable options for optical frequency conversion, to generate new wavelengths and to amplify weak optical signals [27,28].

In the history of nonlinear optics, the discovery of second-harmonic generation (SHG) marked the birth of the field. SHG can be regarded as a wave-mixing process, where an optical wave is mixed with itself, generating a new wave at twice the frequency. As a consequence, part of the energy of the optical wave at frequency ω propagating through the material is converted to that of a wave at 2ω. 

The basic results of two waves mixing in a nonlinear medium is that either the sum (i.e., sum frequency generation (SFG)) or the difference (difference-frequency generation (DFG)) between the input frequencies can be generated. These generations are achieved when a strong pump and a signal are injected into a second-order nonlinear medium and a specific phase-matching condition, selecting only one process, is satisfied. SFG is useful for converting an infrared beam into a more easily detectable visible beam by mixing visible and infrared light or for generating ultraviolet light. In difference-frequency generation (DFG), a weak input optical signal at frequency $\omega_{signal}$ is amplified by a strong laser pump with higher frequency ($\omega_{pump}>\omega_{signal})$, while an idler wave is generated The signal amplification is the fundamental feature of DFG and the most important difference with SFG. Since the signal is amplified by the DFG process, which is a parametric one, DFG is also known as optical parametric amplification. It is worth noting that standard optical amplifiers work only at frequencies corresponding to transitions among specific energy levels, while parametric amplifiers, using transparent crystals, can amplify, in principle, any frequency within the transparency window of the crystal, provided that phase matching can be achieved. In the special case where $\omega_{pump}=\omega_{signal}$, we have the exact reverse of SHG. This process is called degenerate parametric amplification. 

An amplifier becomes an oscillator when a positive feedback is provided, so the parametric amplifier can be transformed into a parametric oscillator by using an optical cavity. In parametric oscillation, when a strong pump $\omega_{pump}$ injects into an optical cavity, including a nonlinear crystal and providing resonance for the signal or the idler (or both), the parametric gain will cause simultaneous oscillations at both the signal and the idler frequencies. The parametric oscillation occurs through amplification of noise photons initiated by parametric fluorescence, and its tuning range can be notably wide. This is of great technical importance as it provides a means for generating intense coherent tunable radiation in the infrared.

**Figure 1:**
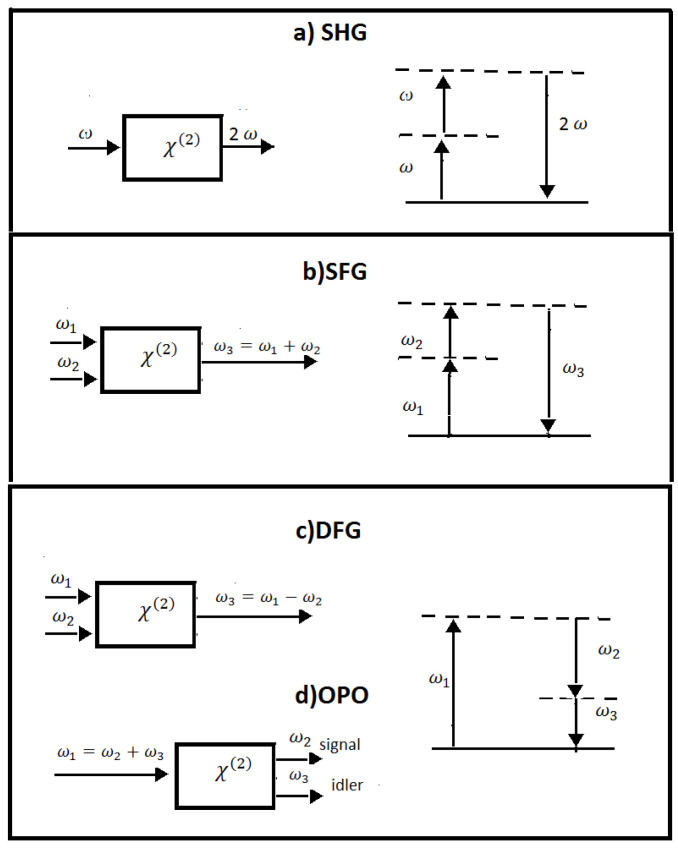
(**a**) Second-harmonic generation, (**b**) sum-frequency generation, (**c**) difference-frequency generation, and (**d**) optical parametric oscillations. In the left column, the geometry of interactions is shown, while in the right column, the energy level diagrams describing the interactions are represented.

Due to the material dispersion of nonlinear media that impacts seriously the phase-matching condition, the second-order nonlinear optical processes, usually, exhibit limited spectral bandwidths. For bulk samples, the phase matching is typically obtained exploiting birefringence in anisotropic nonlinear crystals or with periodically poled nonlinear crystals (e.g., lithium niobate). In such nonlinear crystals, there is a stringent compromise between phase-matching bandwidth and total conversion efficiency, which are inversely and directly proportional, respectively, to the material’s thickness. In addition, the limited availability of high-quality non-centrosymmetric crystalline materials in integrated photonic platforms makes second-order nonlinearities less common as compared with third-order ones [27,28]. In Figure 1, the geometry of interactions and the energy level diagrams of the considered second-order nonlinear phenomena are reported.

### 2.2. Third-Order Phenomena

Third-order effects can be induced by monochromatic and polychromatic fields. In the simplest case, when the applied field is monochromatic, two phenomena are achieved: third-harmonic generation (THG) and intensity-dependent refractive index or Kerr effect.

**Figure 2:**
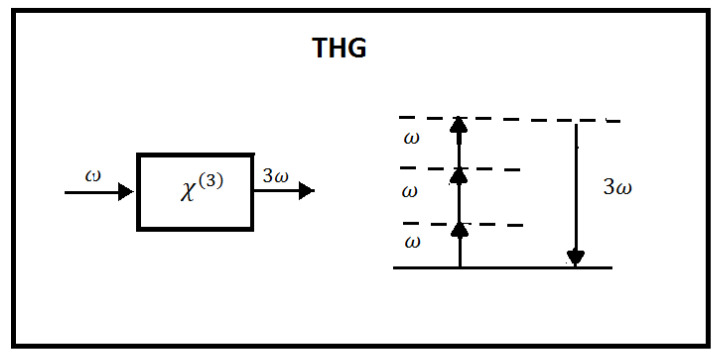
Third-harmonic generation. On the left is the geometry of the interaction, and on the right is the energy level diagram describing the interaction.

THG can be considered as the third-order equivalent of SHG. According to the photon description, in this process, three photons of frequency ω are destroyed, and one photon of frequency 3ω is created. The main application of THG is the realization of ultraviolet (UV) sources, where there are few choices for lasers. However, the practical use of THG requires a material having a large $\chi_{3}$, a transparency window including both $\chi_{1\;}$and $\chi_{3}$, and the possibility of achieving phase matching, too. In Figure 2, the geometry of the interaction and the energy level diagram of THG are reported.

For a monochromatic incident field, third-order effects produce a material polarization: $P_{3}=\varepsilon_{0}\left(\chi_{3}E^{2}\right)E$ that leads to a term in which the factor $\left(\chi_{3}E^{2}\right)$ oscillates at the fundamental frequency of the incident light. This causes the refractive index of materials to be described by $n\left(I\right)=n_{0}+\gamma *I$, where $n_{0}$ stands for the linear refractive index, while the nonlinear refractive index γ is related to the real $\left(Re\;\chi^{\left(3\right)}\right)$ part of the third-order nonlinear susceptibility. The intensity-dependent index of refraction induces the phase effects affecting the beam propagation and leading to a number of related phenomena, i.e., self-focusing, self-phase modulation (SPM), and cross-phase modulation (CPM). All these effects are described by the real part of the susceptibility $\chi_{3}$ and are fundamental for nonlinear photonic applications because they lead to field-dependent modifications of certain material properties, e.g., the refractive index of the medium, which make available a number of devices in which it is possible to control light by light.

Self-focusing is an induced lens effect. Due to the nonuniform transverse intensity distribution of propagating beam, the optical Kerr effect produces a transverse refractive index profile that follows the intensity profile of the beam. If $n_{2}$ is positive, a greater index of refraction is induced on axis than in the wings of the beam, which causes the rays to curve toward each other, creating a positive lens that tends to focus the beam.

**Figure 3:**
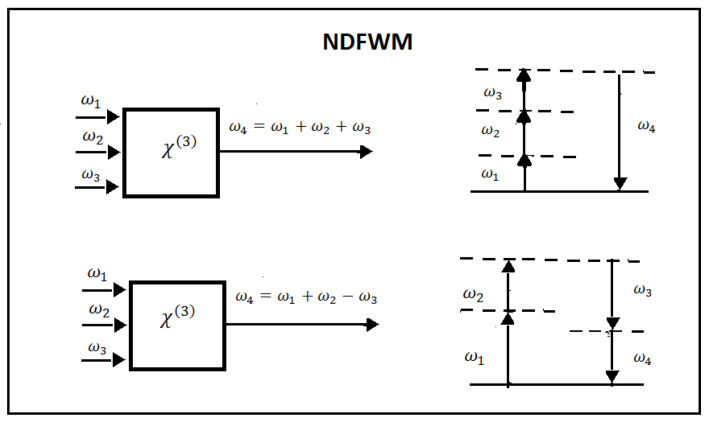
Non-degenerate four-wave mixing. Two of the possible mixing process that can occur when three input waves interact in a medium. On the left are the geometries of the interactions, and on the right are the energy level diagrams describing the interactions.

In the general case, the applied field consists of three different frequency components, and the third-order nonlinear polarization contains a number of contributions with different frequencies. Among them are $\omega_{4}=\omega_{1}+\omega_{2}+\omega_{3}$, $\omega_{4}=\omega_{1}+\omega_{2}-\omega_{3}$, $\omega_{4}=\omega_{1}-\omega_{2}+\omega_{3}$, and $\omega_{4}=-\omega_{1}+\omega_{2}+\omega_{3}$. These represent four possible mixing processes, called non-degenerate four-wave mixing (NDFWM). In basic terms, NDFWM is the mixing of three different frequencies $\omega_{1}\neq \omega_{2}\neq \omega_{3}$, which interact simultaneously with the medium, to produce a fourth wavelength such that the respective frequencies obey one of the previous four equalities. In Figure 3, we represent two of the four possible mixing processes. In Figure 3, interactions and energy level diagrams of two of the possible mixing process are reported.

The conjugated phase of an incident wave can be created by the degenerate FWM. In this process, all the three mixed frequencies have the same frequencies $\omega_{1}=\omega_{2}=\omega_{3}$. They interact in a nonlinear medium, which is illuminated by two strong counterpropagating pump waves and by a signal wave. As a result, a new wave having the same frequency $\left(\omega =2\omega -\omega \right)$ is created that is the phase conjugate of the signal.

**Figure 4:**
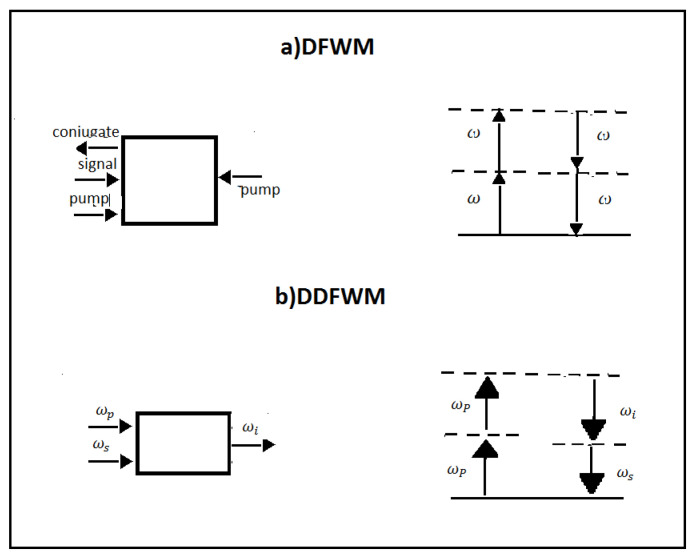
(**a**) Degenerate FWM and (**b**) dual degenerate FWM. On the left are the geometries of the interactions, and on the right are the energy level diagrams describing the interactions.

Note that for the special case of the dual degenerate FWM (DDFWM) when $\omega_{1}=\omega_{2}=\omega_{p}$, two frequencies, related to a pump ($\omega_{p}$) and a weaker signal ($\omega_{s}$), can produce a third one, which is known as the idler frequency, such that $\omega_{i}=2\omega_{p}-\omega_{s}\;$holds. In other words, in the DDFWM, two equal pump photons ($\omega_{p}$) are annihilated to create two output photons called the signal ($\omega_{s}$) and idler ($\omega_{i}$). In Figure 4, interactions and energy level diagrams for the degenerate and dual degenerate FWM are reported. 

We note that in the DDFWM, conservation of energy requires the idler and signal being equally separated from the pump since $\omega_{p}+\omega_{p}=\omega_{s}+\omega_{i}\;\Rightarrow \;\omega_{p}-\omega_{s}=\omega_{i}-\omega_{p}$. In the case of the non-degenerate FWM, the process features two different input photons ($\omega_{p}$ and $\omega_{s}$) and again an idler ($\omega_{i}$) and signal ($\omega_{s^{\prime }}$) photon as the output. In this case, conservation of energy requires $\omega_{p}+\omega_{s}=\omega_{s^{\prime }}+\omega_{i}\;\Rightarrow \;\omega_{p}-\omega_{i}=\omega_{s^{\prime }}-\omega_{s}$, which implies that the signal and idler photons have the same detuning from the two input photons. 

**Figure 5:**
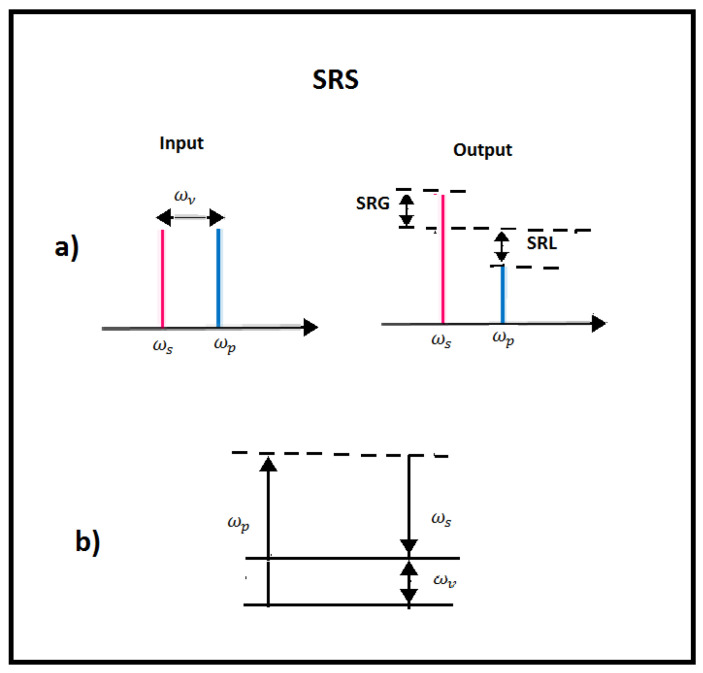
Stimulated Raman scattering. (**a**) SRS modalities: SRG, stimulated Raman gain; SRL, stimulated Raman loss. (**b**) The energy level diagram describing the SRS interaction.

An SRS phenomenon occurs in the presence of a high-energy transfer from a high-power pump beam to a probe beam (copropagating or counterpropagating) [29,30]. In particular, this energy exchange occurs when the frequency difference between the pump and the Stokes laser beams matches a given molecular vibrational frequency of the sample under test. The SRS effect occurs in the form of a gain of the Stokes beam power (stimulated Raman gain (SRG)) and a loss of the pump beam power (stimulated Raman loss (SRL)). SRS depends on the pump intensity and on a gain coefficient. The latter is proportional to the spontaneous Raman scattering cross section and inversely proportional to the linewidth of the corresponding Raman line. Because of its coherent nature, the molecular bonds oscillate in phase and interfere constructively inside the focus area of the laser beam. As a consequence, an SRS signal, which is orders of magnitude bigger than spontaneous Raman scattering, is generated (about 20–30% of the incident laser radiation can efficiently be converted into SRS). Due to its Raman-shifted output, SRS is a workable method for generating coherent radiation at new frequencies. SRS permits, in principle, the amplification in a wide interval of wavelengths, from the ultraviolet to the infrared. Since the Raman frequency of a medium is usually fixed, a tunable pump laser is required to achieve Raman source tunability [31,32]. In Figure 5, the SRG and SRL modalities and level diagrams for SRS phenomena are reported.

In the last two decades, SRS in nanophotonics has received considerable attention [33,34,35,36,37,38,39,40,41,42,43,44,45]. Raman lasers in high-Q resonators have been long investigated because they allow attaining quite low pump threshold powers and high Stokes output powers, when the cavities are designed to have high Q factors at both pump and Stokes wavelengths. A high Q factor over a broad wavelength range is necessary if the Raman lasers are to have a wide tuning range at every resonant wavelength. Single-mode waveguide resonators have good mode confinements for enhanced optical nonlinearities, but they suffer from high propagation losses (typically about 2 dB/cm). On the other hand, conventional multimode racetrack resonators, consisting of one multimode bus waveguide and one multimode racetrack, offer smaller propagation losses for the fundamental modes, but their drawback is the reduction in Q factors of the fundamental mode-based resonances. In ref. [46], a new approach using multimode silicon concentric racetracks, allowing high Q factors to be maintained in every single FSR over a broad optical bandwidth, was presented. Based on this resonator, a widely tunable Raman lasing spanning from 1325 to 1841 nm was experimentally demonstrated [46].

**Figure 6:**
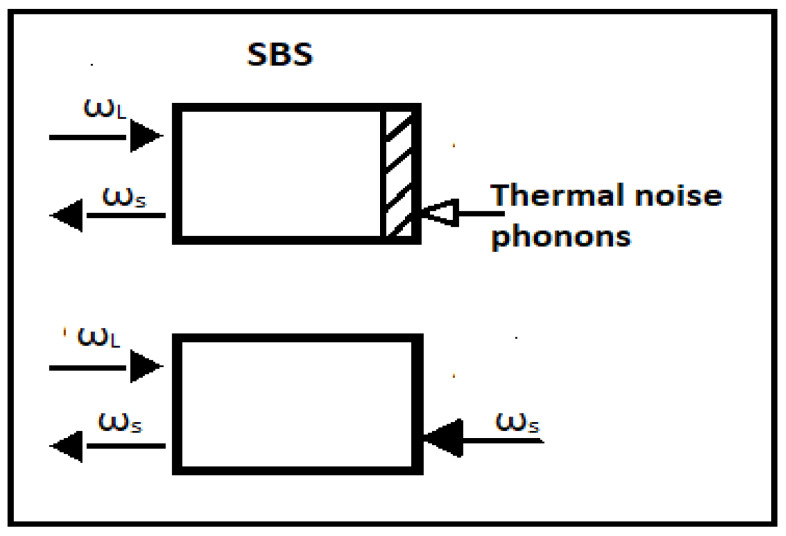
SBS generator on the top and SRS amplifier on the bottom.

Brillouin scattering describes the scattering of an optical wave from an acoustic wave, which can be a longitudinal pressure/density wave in a solid, gas, or liquid; an acoustic surface wave; or a transverse acoustic wave. When an optical wave is scattered from the acoustic wave, a frequency-shifted optical wave is generated, called the Stokes wave, when lower in frequency than the pump wave, and the anti-Stokes wave, when at a higher frequency. This process can be stimulated, which leads to an exponential gain of the optical Stokes wave. A small optical seed that counterpropagates the optical pump creates an optical beat pattern. The small seed can be a laser coupled from the opposite side into the medium than the pump or originate from the scattering of the pump from thermal phonons; see Figure 6. When the frequency separation of the pump and the seed matches the Brillouin frequency shift (BFS) in the medium, then the optical beat pattern reinforces the acoustic wave via electrostriction, which, in return, amplifies the seed/Stokes wave via scattering of the optical pump from the moving acoustic wave. This feedback can create strong amplification of the initially weak seed/Stokes wave [47]. In Figure 6, level diagrams for SBS phenomena are reported.

A new trend of Brillouin research focuses on integrated photonic platforms. Microscale waveguides, engineered to guide acoustic and optical waves, enable new ways to control and manipulate optical signals and promise many novel applications in a compact and small footprint. Recently, two parallel trends for chip-scale platforms emerged: (1) SBS in high-Q resonators to enable SBS enhancement and (2) low-loss on-chip waveguides that guide both optical and acoustic modes. In the optical domain, SBS is used to achieve functionalities such as non-reciprocity and optical isolation, nonlinear mode conversion, delay and storage of optical signals, and ultra-narrowband lasing in resonators. A deeper understanding of the fundamental interactions between light and sound has drawn substantial interest in recent years toward optomechanical cavities that aim to address fundamental scientific questions, for example, the observation of quantum effects in macroscopic systems [48].

In nonlinear regimes, the optical absorption of materials is described by $\alpha \left(I\right)=\alpha_{0}+\delta *I$, where $\alpha_{0}$ stands for the linear absorption, while nonlinear absorption δ is related to the imaginary $\left(Im\;\chi^{\left(3\right)}\right)$ of the third-order nonlinear susceptibility. Nonlinear absorption phenomena refer to the change in transmittance of a material as a function of intensity. They involve two-photon absorption (TPA), saturable absorption (SA), or multiphoton absorption (MPA) [24,25].

**Figure 7:**
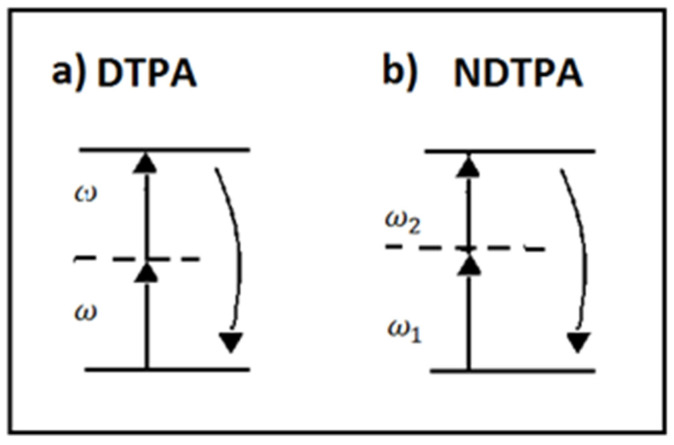
The energy level diagram describing the interactions: (**a**) degenerate TPA and (**b**) nondegenerate TPA.

Two-photon absorption (TPA) is a third-order nonlinear optical interaction, in which two photons are simultaneously absorbed through a virtual intermediate state to produce a real excitation. We can distinguish between two types of TPA: a degenerate process, in which two photons with the same wavelength are absorbed, and a nondegenerate process, in which one photon with wavelength λp from a pump source and one photon from a signal source with wavelength λs are both absorbed. The latter can be applied to modulate a probe signal through a process called cross-absorption modulation (XAM). Nonlinear absorption and related phenomena such as two-photon absorption and saturable or reverse saturable absorption have some important applications in light generation (Q-switching and mode-locking lasers), in laser modulation, taking advantage of cross-modulation (XAM), and in nonlinear detection of light by TPA. In Figure 7, level diagrams for DTPA and NDTPA are reported.

In contrast to the TPA process where the absorption increases with light intensity reverse saturable), SA exhibits the opposite trend. Due to the saturation of excited electrons filling the conduction band and hence preventing further transitions due to Pauli blocking, in saturable absorption, when the frequency of incident light is near an absorption resonance of the material, as the intensity increases, an absorption reduction is obtained. The SA is useful for applications such as mode-locked fiber lasers and all-optical modulators.

Multiphoton absorption is a process in which an atom or molecule undergoes a transition from a ground state to an excited state by means of the simultaneous absorption of N photons. Multiphoton transitions take place via “virtual” energy levels. This phenomenon can be used for the three-dimensional microfabrication of photonics devices.

### 2.3. Ultrafast Phenomena

Nowadays, photonics is the technology of choice for the transmission and routing of vast amounts of very high-speed data through optical fibers [49,50]. The fabrication of low-loss single-mode optical fibers has made possible optical communication links with demonstrated bandwidths exceeding several terahertz, which are used to transmit data over hundreds of kilometers, without the need of any regeneration stage. As the demand for bandwidth increases, communication systems are forced to use higher bit rates and, hence, to require shorter pulses, for which waveguide dispersion tailoring becomes fundamental.

We note that even for the case of the medium with a linear response, the shape of a laser pulse can be modified by means of propagation effects, such as dispersion of the group velocity within the medium. Given a single input pulse at the carrier frequency $\omega_{0}$, each spectral component of the input field propagates as a plane wave and acquires a slightly different phase shift because of the frequency dependence of the propagation constant β. It is useful to expand β in a Taylor series around the carrier frequency $\omega_{0}$, and depending on the pulse bandwidth, the second-order dispersion term ($\beta_{2}$), called the group velocity dispersion (GVD), and the third- or higher-order dispersion terms must be considered. After transmission, even if the initial pulse is unchirped, the transmitted pulse becomes chirped, and the sign of linear chirp depends on the sign of GVD; if $\beta_{2}>0$, an up chirp is obtained and a broadening of pulse in time is achieved, while if $\beta_{2}<0$, a down chirp is obtained. The latter can be used for the compression of laser pulses. 

**Figure 8:**
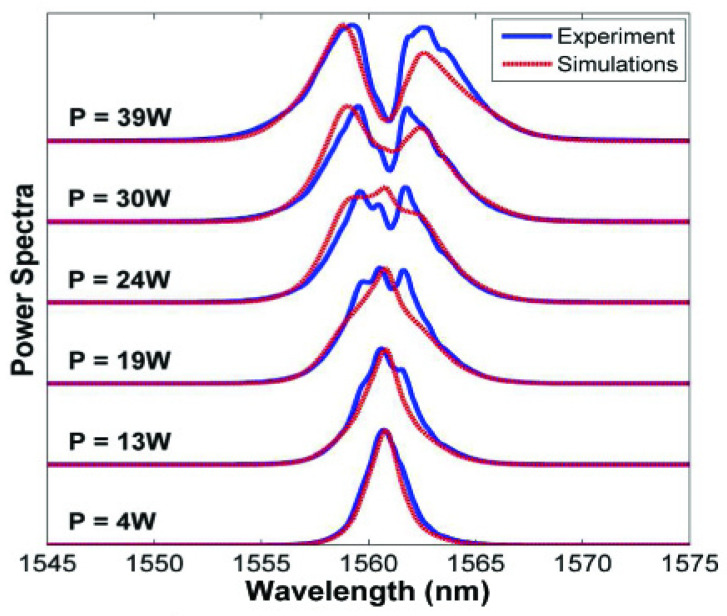
Efficient self-phase modulation, using ~1 ps pulses near 1560 nm, was achieved in low loss (<0.06 dB/cm) and 45 cm long spiral integrated waveguide of high-index silica glass [49]. In this figure, experimentally measured output power spectra (solid blue lines) and theory (dashed red lines) for different input (coupled) power levels are reported. Reprinted with permission from [52] © The Optical Society.

When short optical pulses propagate through third-order nonlinear optical media and the dispersive term can be neglected, their spectral content can be modified by a nonlinear optical process named self-phase modulation The optical Kerr effect produces a time-dependent change in the refractive index, which induces a time-variable phase shift φ of the propagating wave. As a result of the of nonlinear varying phase, the spectrum of the transmitted pulse will be typically broader than the incident pulse, i.e., new frequency components are created, keeping the temporal shape unaltered [49,50,51,52]. As an example, in Figure 8, self-phase modulation achieved in high-index silica glass is reported.

The intensity dependence of the refractive index leads to another nonlinear phenomenon known as cross-phase modulation (CPM). When two or more optical pulses propagate simultaneously, the cross-phase modulation is always accompanied by SPM and occurs because the nonlinear refractive index seen by an optical beam depends not only on the intensity of that beam but also on the intensity of the other copropagating beams. In fact, CPM converts power fluctuations in a particular wavelength channel to phase fluctuations in other copropagating channels. The result of CPM may be an asymmetric spectral broadening and a distortion of the pulse shape. The CPM phenomenon can be used for optical switching, using interferometric methods. In an interferometer, a weak signal pulse can be divided equally between two arms, it experiences identical phase shifts in each arm, and it is transmitted through constructive interference. When a pump pulse at different wavelength is injected into one of the arms, it will change the signal phase through CPM phenomenon in that arm. If the CPM-induced phase shift is large (close to π), this phase shift results in destructive interference and hence no transmission of signal pulse [49,50].

**Figure 9:**
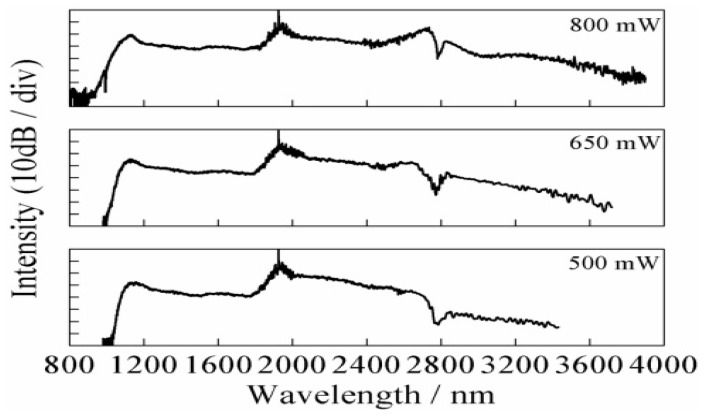
Measured SC in a tellurite microstructured optical fiber (MOF) at the pump wavelengths of ~1958 nm with average pump powers of ~500, 650, and 800 mW. With the average pump power increasing to ~800 mW, the broadband mid-infrared SC generation with the spectrum from ~900 to 3900 nm is observed. Reprinted with permission from [54] © The Optical Society.

An interesting nonlinear phenomenon is supercontinuum (SC) generation [53,54]. An ultrabroadband emission is produced by a material upon irradiation with an intense laser source, due to a complicated interplay among dispersive term and NLO processes, such as self-phase modulation, stimulated Raman scattering, and soliton effects [50,51]. In Figure 9, as an example, measured SC in a tellurite microstructured optical fiber (MOF) is reported.

In optics, an optical wave packet (a pulse or a beam) has a natural tendency to spread as it propagates in a medium, due either to chromatic dispersion or to spatial diffraction. When this natural broadening is compensated by a nonlinear process, a stable self-localized wave packet is formed. Such a self-trapped wave packet, whether in time or space or both, is known as an optical soliton. Solitons are localized wave entities that can propagate in nonlinear media, exhibiting invariant or recurrent propagation behavior. This means that the mutual compensation of dispersion and nonlinear effects can take place continuously with the distance as in the case of “classical” solitons or periodically as in the case of dispersion-managed solitons [55,56].

The temporal broadening of a pulse in a dispersive material due to chromatic dispersion, i.e., linear group velocity dispersion (GVD), can be compensated by the narrowing associated to self-phase modulation (SPM), so that a narrow pulse can propagate without temporal spreading. We note that when the normal dispersion is positive ($\beta_{2}>0$), both the pulse shape and spectrum change as the pulse propagates through the fiber/waveguide, and the combined effects of GVD and SPM cause a broadening of the pulse both in time and frequency. Whereas for anomalous dispersion ($\beta_{2}<0$), where $\beta_{2}$ and $n_{2}$ can have opposite signs, the group velocity dispersion induces a down chirp, while SPM nonlinearity induces an up chirp, so these two effects can compensate each other. This could be surprising since GVD affects the pulse in the time domain, while the SPM effect is in the frequency domain. However, a small time-dependent phase shift added to a Fourier transform-limited pulse does not change the spectrum to first order. If this phase shift is canceled by GVD in the same fiber, the pulse does not change its shape or its spectrum as it propagates [55]. Temporal solitons have been observed in laser resonators [57], microresonators [58,59], and microcombs [60]. In each of these cases, nonlinear compensation of GVD is provided by the Kerr effect.

Optical spatial solitons are shape-invariant self-guided beams of light, i.e., their beam diameter remains invariant during propagation in nonlinear media, thanks to a dynamical balance between diffraction and a self-focusing effect. In other words, a laser beam can produce its own dielectric waveguide and propagate without spreading. To obtain this result, it is necessary to compensate the spatial broadening of the beam due to diffraction during the propagation with the self-focusing. At low intensities, the nonlinear Kerr effect can be neglected, and the beam spreads under diffraction inducing a positive wavefront curvature. In contrast, an intense single beam with a well-shaped intensity profile at its waist generates a refractive index distribution looking like a graded index thick lens when the n_2_ coefficient is positive, and as a consequence of balance with diffraction, a constant transverse dimension of the beam during propagation is maintained. An analysis of this phenomenon shows that it occurs when the power carried out by the beam is exactly equal to the critical power for self-trapping [61,62]. As an example, Kerr spatial solitons in slab chalcogenide waveguides at near-IR wavelengths have been observed [63].

**Figure 10:**
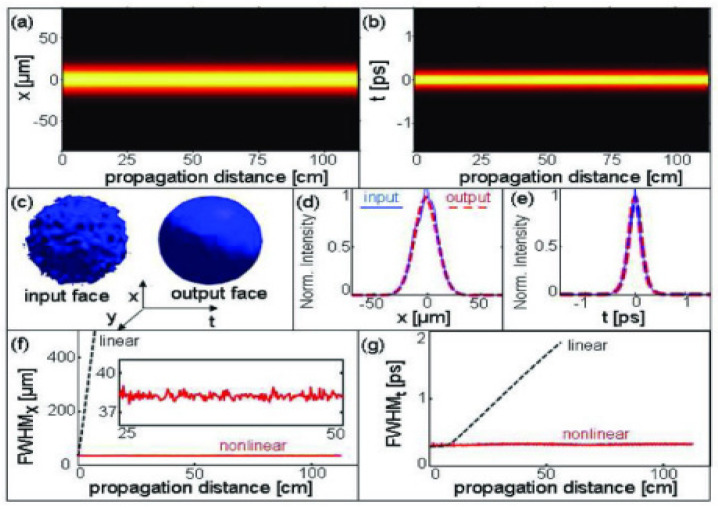
Temporally bright spatiotemporal pulse-train soliton. Intensity of the soliton in plane xz (**a**) and t-z (**b**) demonstrating the stationary propagation. (**c**) Intensity in x-y-t at the input and output faces of the sample. Intensity profiles in the x direction (**d**) and t direction (**e**) at the input and output faces of the sample. Width (FWHM) of the intensity in the x direction (**f**) and t direction (**g**) versus propagation distance for linear and nonlinear propagations. Reprinted with permission from [67] © The Optical Society.

Distortionless propagation, i.e., a mechanism capable of holding together the beam in the space and time domain, has a great technological relevance for optical telecommunications [55,58]. The use of fundamental solitons as an information bit can solve the problem of dispersion because in a fiber channel, nonlinear phase modulation can compensate for linear group dispersion leading to pulses that propagate without changing the temporal shape and spectrum. Chip-scale devices that support optical solitons harness high field confinement and flexibility in dispersion engineering for significantly smaller footprints and lower operating powers compared with fiber-based equivalents [64,65,66,67]. In Figure 10, a temporally bright spatiotemporal pulse-train soliton is reported as an example [67].

## 3. Photonic Nonlinear Materials

In this section, we classify photonic nonlinear materials in three groups: semiconductors, glassy material and lithium niobate, and innovative materials.

### 3.1. Semiconductors

In the last decades, silicon photonics has received considerable attention. The main advantage of silicon photonics has been its compatibility with mature CMOS technology, offering structure sizes down to 10 nm at low cost. Silicon photonics has proven to be a promising technology (and in some cases a very effective solution) for a variety of applications; data center transceiver application has been the most successful, but consumer health and photonic computing are now emerging as rapidly growing fields. Other important applications, such as free-space communication, chemical and biomolecular sensing, and infrared spectroscopy, would push the development of integrated photonics toward the mid-infrared region, but another shortcoming of SOI platform is related to the loss of the buried oxide layer of the SOI waveguides at mid-infrared wavelengths. Silicon photonics also extends to highly integrated multifunctional devices that perform both optical and electrical operations on a single low-cost chip [68,69,70].

On the other hand, the major challenge in the development of active silicon elements is due to the indirect bandgap of silicon, which makes spontaneous emission unlikely and thus prevents lasing. This physical limitation with respect to light emission is a serious drawback when compared with integrated optical devices based on III-V direct bandgap materials, which are capable of efficiently emitting light. Overcoming this limitation in silicon is considered to be the holy grail of silicon photonics. Unfortunately, for a variety of reasons, room temperature electrically injected silicon-based lasers remain elusive to date. Another important shortcoming is that silicon does not absorb light at wavelengths above the material’s bandgap (∼1.1 μm), so light detection is prevented in the range of interest for telecommunications [68,69,70,71,72].

When going to nonlinear optical applications, several shortcomings are long known, such as the high optical absorption at telecommunication wavelengths and the inherent lack of second-order optical susceptibility ($\chi^{\left(2\right)}$) due to Si centrosymmetric lattice structure. The latter, of course, limits the use of Si photonics for standard applications, such as the development of electro-optic silicon modulators based on second-order nonlinear effects. In recent years, many types of third-order nonlinear optical phenomena have been investigated in silicon-based photonic devices. Substantial progress has been achieved, e.g., in the field of Raman amplification, while nonlinear effects such as two-photon absorption (TPA), self-phase modulation (SPM), cross-phase modulation (XPM), continuum generation, four-wave mixing (FWM), and the optical Kerr effect have also been successfully demonstrated and thoroughly investigated, on time scales ranging from the femtosecond to the nanosecond regime. However, even if silicon has large Si third-order optical susceptibility ($\chi^{\left(3\right)}$) compared with silica fibers or a silica integrated optical platform [73], the exploitation of silicon photonics devices is limited, at least at telecom wavelengths, by the presence of two-photon and free-carrier absorptions (TPA and FCA), at the required high optical intensities [68,69,70,71,72,73,74].

In order to address the shortcomings of silicon photonics, several materials have been investigated based on the heterogeneous integration of other material systems on silicon substrates, with the common requirement to remain compatible with the complementary metal-oxide-semiconductor (CMOS) technology [75]. The typical materials adopted for these heterogeneous integrations include GeSi, Ge-on-Si, silicon nitride (SiN), amorphous silicon (a-Si), silicon nanocrystal (Si-nc) [34,35,36,37,38,39,40,41,42], and silicon carbide (SiC). While germanium’s cutoff wavelength of ~1.8 μm appeals as a perfect choice, in order to realize photodetectors, SiN, a-Si, Si-nc, and SiC are considered as appealing material for nonlinear photonic applications. 

SiN has been routinely used in fabrication processes and final device structures for electronic integrated circuits. Due to its wide transparency window from visible to mid-infrared, progress on advanced SiN fabrication processes has enabled ultra-low propagation losses in waveguide structures. Due to the larger energy bandgap, SiN shows negligible TPA in the telecom bands, while the Kerr coefficients (n_2_) of stoichiometric and non-stoichiometric SiN are about an order of magnitude and about three times smaller than silicon [76], respectively. However, the combination of linear and nonlinear optical properties makes photonic signal processing based on silicon nitride a promising research area. Silicon-rich nitride waveguides for ultra-broadband nonlinear signal processing (e.g., wavelength conversion and radio-frequency spectrum analyzer) have been demonstrated [77,78]. Recently, large second-harmonic generation enhancement in Si3N4 waveguides has been also demonstrated [79,80].

A drawback of silicon-rich and ultra-silicon-rich nitride films, realized by CVD, is the presence of N–H bonds and Si–H bonds, which possess absorption overtones close to 1.55 μm. In order to reduce Si–H related loss, precursor gases, which do not contain any Si–H bonds, can be considered. Deuterated silane (SiD_4_) is chemically almost identical to SiH_4_, but the Si–D bonds have an absorption overtone close to 2 μm. In ref. [81], the growth and optical characterization of silicon-rich nitride films grown using SiD_4_ gas were reported. The absence of Si–H bonds and a reduction of propagation losses in waveguides compared with a conventional SRN film were demonstrated. A nonlinear refractive index of 980 × 10^−20^ m^2^ W^−1^, about two orders of magnitude larger than in stoichiometric silicon nitride, was demonstrated. The bandgap of 1.9 eV indicates that two-photon absorption is absent at a wavelength of 1.55 µm.

Amorphous silicon (a-Si) has been identified as a possible solution to overcome the main limitations showed by the SOI platform. Indeed, a-Si shows enhanced nonlinear performance with respect to crystalline silicon, exhibiting an enhanced Kerr response. The Kerr coefficient (n2) is about an order of magnitude greater than that of silicon [82,83,84]. 

Silicon carbide (SiC) exhibits unique optical properties that can be utilized for novel photonic devices. SiC is a wide bandgap compound semiconductor, so it is a transparent material from the UV to the infrared, and it is a meta-material in the mid-infrared range. Improving the quality factor (Q) of the microring and microdisk resonators in the SiC on insulator (SiCOI) platform, NLO effects based on high second-order and third-order nonlinear coefficients have been recently observed also by nanostructuring SiC [80,81]. The third-order nonlinearity n_2_ of 60 × 10^−20^ m^2^/W at 1550 nm for 4H-SiC [85,86] was reported, while in a-SiC, a value of the Kerr nonlinearity of n_2_ = 480 × 10^−20^ m^2^/W was estimated [87], which is of the same order of magnitude as silicon. The unique combination of excellent electronic, photonic, and spintronic properties of SiC has prompted research to develop novel devices and sensors in the quantum technology domain, too [87,88,89].

Tantalum pentoxide (Ta_2_O_5_) is a CMOS-compatible material that has been very recently considered as a possible alternative for nonlinear applications at telecom-wavelength applications. Thanks to its extremely large bandgap value (3.8 eV), no TPA effect is present at the 1550 nm wavelength region, while its n_2_ is an order of magnitude lower than that of silicon [90,91,92].

Among the III–V integrated nonlinear photonic platforms considered to date, AlGaAs is the most studied waveguide platform [93]. AlGaAs has been termed “the silicon of nonlinear optics” for a number of reasons. The bandgap energy of AlGaAs can be modified by altering the aluminum concentration. It exhibits a wide transparency window from near- to mid-infrared (MIR, 0.9–17 μm), low linear and nonlinear propagation losses in the telecom spectral range (1400–1600 nm), a strong electro-optic effect, a large thermo-optic coefficient (two times larger than that of Si) enabling efficient thermal tuning, and a large second-order nonlinear susceptibility (χ^(2)^ over 200 pm/V) and third-order optical nonlinearity (Kerr coefficient n_2_ = 1500 × 10^−20^ m^2^/W) [94,95]. Depending on the structure and the composition, values of the nonlinear refractive index n_2_ measured in (Al)GaAs waveguides range from 210 × 10^−20^ m^2^/W at 1600 nm in an Al_0.23_Ga_0.77_As ridge waveguide [96] up to 5500 × 10^−20^ m^2^/W at 1505 nm in a GaAs/AlAs structure constituting six layers (a 0.6 µm thick GaAs/AlAs superlattice core layer with 75 periods of 14:14 monolayers each, two 0.3 µm thick Al_0.56_Ga_0.44_As buffer layers, and two Al_0.60_Ga_0.40_As cladding layers (the upper being 0.8 µm thick and the lower 4.0 µm, with a 0.1 µm GaAs cap) [97]. The n_2_ value of that multilayer structure is impressive; for comparison, in a graphene/Si hybrid waveguide, the effective Kerr coefficient n_2_ was calculated to be ~2000 × 10^−20^ m^2^/W, five times higher than that of the Si waveguide alone [98].

The advances in the fabrication process made it possible to minimize the propagation loss in AlGaAs waveguides, e.g., in the AlGaAs-on-insulator (AlGaAs-OI) material platform, down to values less than 1 dB/cm, not so different from those achievable in SOI waveguides [93]. To date, in AlGaAs waveguide platforms, second- and third-order nonlinear optical phenomena, such as SHG, SFG, DFG, spontaneous parametric down-conversion (SPDC), FWM, 2PA, SPM, XPM, spontaneous four-wave mixing (SFWM), spatial soliton formation, SRS, and supercontinuum generation (SCG), have been experimentally investigated. AlGaAs waveguide platforms can be used for a wide range of integrated nonlinear photonic devices, including all-optical signal processing in optical communication networks and Kerr frequency microcomb and integrated quantum photonic circuits.

Recently, efforts have been made to realize photonic devices in gallium phosphide (GaP). The motivations were (1) GaP is nearly lattice-matched to silicon, in principle enabling wafer-scale production; (2) GaP has negligible two-photon absorption (TPA) for wavelengths above 1.1 μm; (3) among visibly transparent III–V materials, GaP has the largest refractive index (n_0_ > 3), enabling strong optical confinement and implying a large χ^(3)^ nonlinearity [n_2_ = 1.100 × 10^−20^ m^2^ W^−1^]; and (4) the non-centrosymmetric crystal structure of GaP yields a large χ^(2)^ nonlinearity. Thus, a variety of GaP nanophotonic devices have been fabricated and studied, including one-dimensional (1D) and 2D photonic crystals, microdisks, and strip waveguides. Among the different applications, particular attention has been paid to realizing frequency doublers from telecommunication to visible wavelengths and solid-state quantum emitters [99].

A crucial point is that GaP must be integrated onto a low index material—ideally by a method compatible with wafer-scale production—then patterned into devices with sufficiently low propagation loss to permit net optical gain. In ref. [99], a GaP-on-insulator platform for integrated nonlinear photonics was proposed, making use of direct wafer-bonding to integrate high-quality, epitaxially grown GaP onto SiO_2_. The large index contrast between GaP and SiO_2_, in conjunction with highly anisotropic, low-roughness dry etching, allowed the realization of single-mode strip waveguides with low propagation losses. As a demonstration of the capabilities of the platform, frequency comb generation in GaP microresonators with a threshold power as low as 3 mW and with the simultaneous formation of doubled combs at visible wavelengths was observed [99].

### 3.2. Glassy Materials and Lithium Niobate

Glasses are interesting non-linear optical materials, being isotropic and transparent in a wide spectral range, combining a low cost of fabrication with high optical quality, manufacturable not only as bulk shapes, or fibers, but also as thin films (e.g., nonlinear planar waveguides) [100,101]. The peculiar characteristics of glass materials are that their optical properties can be adjusted through doping and compositional changes to fit the specified requests of each application. Glasses are non-crystalline (or amorphous) materials with short-range order, so due to their isotropic structure, they have inversion symmetry and do not exhibit second-order nonlinearity, χ^(2)^, or Pockels effect. However, second-order nonlinearity can be achieved by an appropriate modification, obtained, for example, by the application of both heat and electric fields (thermal poling). In silicate glasses, χ^(2)^ also appeared by the introduction of optical non-linear nanocrystals within a glass matrix, obtained by precipitation of crystallites of non-centrosymmetric compounds [102]. This strategy gives rise to transparent crystallized glasses (glass-ceramics). The nonlinear optical properties of glasses have been used in several technological applications with a broad spectrum of phenomena, such as optical frequency conversion, optical solitons, phase conjugation, and Raman amplification [43,44,45,46].

The optical fiber based on fused silica glass is one of the most important platforms for guided-wave optics. For its excellent properties including extremely low losses between ~0.2 and 2 μm, low dispersion, and low nonlinearity, it has been the backbone of the contemporary telecom infrastructure. Due to the low Kerr coefficient [103], standard step-index fused silica fiber is not the most suitable for nonlinear optics. To overcome these limitations, new glasses for optical device applications and photonics have been investigated. These include fluoride glasses, tellurite glasses, aluminosilicates, phosphate glasses, borate glasses, and chalcogenide glasses. Glasses based on heavy metal oxides, such as Sb, Bi, Pb, W, Ga, Ge, and Te, allow applications such as optical switches due to their characteristics of low linear and nonlinear loss, large Kerr nonlinearity, and ultra-fast response. High-index glass (Hydex™) is a special type of doped fused silica glass with a refractive index in the range from 1.5 to 1.9. It has been used as an alternative material for CMOS-compatible low-loss optical waveguides, but it has a relatively low Kerr nonlinearity [52,104]. Chalcogenide glasses are formed by the chalcogen elements S, Se, and Te and the addition of other elements such as Ge, As, Sb, Ga, etc. They have excellent transparency in the mid-IR region and are relatively easy to process, so they are the basis for the manufacture of devices operating in the mid-infrared region, where conventional silica glass shows strong absorption. Chalcogenide glass is one of the most interesting materials for nonlinear photonics since it possesses large optical nonlinearities in the infrared spectrum [105,106].

The field of third-order nonlinear phenomena of glasses has been mainly focused on two main groups: resonant and non-resonant [100,101]. Non-resonant phenomena occur when the light excitation falls in the transparent wavelengths range of the glass longer than its electronic absorption edge. As no electronic transitions take place, the process can be seen as lossless, and an ultrafast glass response due to third-order electronic polarization is assured. Examples are, in general, high-refractive-index and high-dispersion glasses such as heavy metal oxide glasses or chalcogenide glasses. Resonant phenomena occur when the frequencies of optical field are near the electronic absorption edge. Metallic nanoparticle doped glasses and semiconductors nanocrystals (quantum dots (QDs)) such as CdS, CdSe, CdTe, PbS, CuCl, etc. doped glasses are suitable materials for resonant NLO devices with response times on the ps domain. Great interest has driven the study of the third-order nonlinear susceptibility of metal particles embedded in dielectric matrices, such as glasses [107], which are influenced not only by the type and size of the metal particles but also by the metal-dielectric constant. The most significant effect of the confinement of metal particles on the optical properties of nanocomposite glasses is the appearance of the surface plasmon resonance, which deeply enhances the glass χ^(3)^ responses with picosecond temporal responses. QD-doped glasses can be prepared through the dispersion of a nanocrystalline phase in a glass matrix. This approach, through the reduction of bulk size to nanometric scale or quasi-zero-dimensional quantum dots, allows the change of the electronic properties of glasses accordingly with enhanced nonlinearity compared with the corresponding bulk semiconductors [100,101].

Lithium niobate (LN) is a transparent material in a wide wavelength range from 0.5 to 4 μm. Due to the intrinsic birefringence, its large EO coefficient (r_33_ = ≈31 pm/V and r_13_ = 8 pm/V), and the capacity of modulation bandwidths in excess of 100 GHz, LN has been recognized for a long time in the photonic industry as the best material for EO modulation. Having a non-centrosymmetric structure, LN shows both $\chi^{\left(2\right)}\;$and $\chi^{\left(3\right)}$ optical nonlinear responses. Lithium niobate has a high second-order optical nonlinearity (d_33_ = −33 pm/V at λ = 1.064 μm) enabling parametric wavelength conversion and optical signal generation [105,106]. Due to its ferroelectricity, LN can be periodically poled (PPLN) with alternate domains of inverted electric dipole orientation to meet efficient quasi phase-matching conditions [108,109,110,111,112,113,114,115,116].

Table 1 presents the nonlinear refractive indices n_2_, two-photon absorption coefficients $\beta_{TPA}\;$and figures of merit FOM of various materials. The wavelength at which the measurement had been made is also indicated.

### 3.3. Innovative Materials

Most photonic applications would strongly benefit from tunable and reconfigurable properties of materials. In order to deal with this challenge, recently, phase-change materials (PCMs) have become a popular method of optical tunability without any moving parts. PCMs are a class of materials with unique physical properties: their structural arrangement can be controllably modified back and forth on a fast timescale using a thermal, electrical, or optical excitation [117,118]. For some of these materials, the crystallographic re-arrangement translates into a large refractive index modification (Δn ≥ 1). Such a large and fast refractive index modulation is a long-sought effect for photonics: an enabling technology to control and tune in real time the optical properties of devices at the nanoscale. Among PCMs, vanadium dioxide (VO_2_) is a prototypical example of functional materials showing large modifications in their physical properties upon specific external excitation [119].

Research in 2D layered materials (2DLMs) has started with the discovery of graphene [120]. The 2DLMs commonly refer to crystals composed of few layers of atoms, whose electrons move freely only on two dimensions (scale: 1–100 nm). It has been realized that the van der Waals layered materials with atomic thickness can not only exist stably but also exhibit unique and technically useful properties including small size effect, surface effect, macro quantum tunnel effect, and quantum effect. The 2D materials meet several requirements of an ideal nonlinear optical material: large and ultrafast nonlinear optical response, ultrafast photoexcitation dynamics, broadband and tunable optical absorption, saturable absorption characteristics, ultrafast recovery time, strong interlayer coupling, and large optical and thermal damage threshold [120,121,122,123,124,125,126,127,128,129,130,131,132,133,134,135,136,137,138,139,140,141,142,143,144]. The 2DLMs have been successfully employed in all-optical modulators, as broadband efficient and versatile saturable absorbers in passive mode locking and Q-switching, in wavelength converters, and in optical limiters [144,145,146].

The remarkable properties of 2D forms of graphite have attracted a rapidly growing research interest. In graphene, the conduction band and valence band converge at the Dirac point, which indicates that graphene owns a gapless semimetallic band structure. However, graphene is widely studied due to its broadband absorption at optical and terahertz (THz) frequencies [121,122,123,124]. Graphene exhibits saturable absorption behavior [125,126], which plays an important role in lasing applications and optical limiting devices.

Recently, graphene oxide (GO), with its large optical nonlinearity, high flexibility in altering its properties, facile solution-based synthesis process, and high compatibility with integrated device fabrication, offers competitive advantages for industrial manufacturing beyond the laboratory, which is a challenge for the majority of 2D materials.

The bandgap of GO can be engineered, and its value spans between 2.1 and 3.6 eV, yielding an absorption that is over two orders of magnitude lower than that of graphene in the telecom band. Similar to graphene, GO films exhibit strong anisotropy in their optical absorption in a broad band from the visible to the infrared regions. This property is useful for implementing polarization selective devices with wide operation bandwidths. Due to heterogenous atomic structure, GO exhibits noncentrosymmetry, yielding a large second-order optical nonlinearity, which is absent in pristine graphene that has a centrosymmetric structure. We note that at the moment, applications of second-order optical nonlinearity to chip-scale devices are still in their infancy.

The absolute value of n_2_ for GO is about 10 times lower than that of graphene, but it is about four orders of magnitude higher than that of nonlinear bulk materials such as silicon and chalcogenide glasses. Enhanced FWM in GO hybrid integrated devices was demonstrated using Hydex waveguides [57] and Si_3_N4 waveguides. Most of the state-of-the-art GO nonlinear integrated photonic devices incorporate GO films with little modification or optimization of their properties. However, GO’s properties can be significantly changed, offering a high degree of flexibility in engineering its capabilities for different nonlinear optical processes [127,128].

Other classes of recently discovered 2DLMs include transition metal chalcogenides (TMCs) [129,130], transition metal-dichalcogenides (TMDs, e.g., MoS_2_) [131,132,133,134,135,136,137], black phosphorus (BP) [138,139,140], and hexagonal boron nitride (h-BN) [141,142], which have broadened the set of specific properties and functionalities of 2D materials.

TMCs are semiconductor materials with the formula MX, where M represents the transition metal from group 4 to 7 (such as Mo, W, Re, and Ga) and X is the chalcogenide element, which is usually one of the three elements S, Se, and Te. TMCs exhibit the typical layer-dependent tunable bandgap, spanning from 1 eV for bulk or ML cases to 3 eV for single-layer (SL) configurations [131,132]. Transition metal dichalcogenides (TMDs) have the formula MX_2_ (such as MoS_2_, MoSe_2_, MoTe_2_, WS_2_, WSe_2_, and TiS_2_). The single-layer TMD structure can be roughly represented by the three-layer covalent bonding in the form of X–M–X, in which metal atoms M are sandwiched between two layers of chalcogen atom X, and the chemical bond M–X provides the connection endowing the whole layer with strong stability, the thickness of which is usually 6–7 Å. Compared with graphene, the 2D TMDs are more suitable for optical applications due to their direct and tunable bandgaps and relatively high carrier mobility [131,132]. The inherent 2D and semiconductor character of the TMDs inevitably lead to the excitonic effects, typical of the optical response of the semiconductors. The optical properties of the 2D MoS_2_ material are sensitive to the number of layers and interlayer distance, which makes it difficult to anticipate the optical properties of these materials. Several theoretical and experimental investigations have proved that TMDs (e.g., MoS_2_ and WS_2_) exhibit nonlinear optical response well beyond their lower energy bandgap (~1 eV) [133,137].

BP is a thickness-dependent direct bandgap semiconductor that could be widely tunable from 0.3 eV (bulk) to 2 eV (single layer (SL)). It bridges the gap between zero bandgap graphene and relatively wide bandgap TMCs, making it suitable for broadband optoelectronic applications, particularly in the IR and mid-IR regions [138,139,140].

Hexagonal boron nitride (h-BN) is a graphene analogue having a crystallographic appearance with boron and nitrogen atoms in place of carbon. h-BN has a large bandgap of ~6 eV. BP also exhibits saturable absorption followed by an optical limiting response upon varying the pump fluence. It finds nonlinear optics applications in UV to NIR regions [141,142].

According to their predominant electromagnetic response, materials or structures with near-zero parameters at a given frequency can be classified as epsilon-near-zero (ENZ), ε≅0; mu-near-zero (MNZ), μ≅0; and epsilon-and-mu-near-zero (EMNZ), ε≅0 and μ≅0 media. All aforementioned classes exhibit a near-zero index of refraction$\;n=\sqrt{\varepsilon \mu }≅0$ at the frequency of interest and can be jointly addressed as zero-index media [147,148,149,150]. These materials or structures empower two of the main requirements to boost the nonlinear response of matter: phase matching [150] and high field intensities. Structures with near-zero parameters can provide large field intensity enhancements over large regions, while simultaneously providing phase matching [147,148,149,150]. ENZ materials are becoming a platform to obtain non-classical, non-reciprocal, and non-local responses of matter [147,148,149,150]. ENZ structures loaded with nonlinear media are particularly well suited to enable the coherent superposition of nonlinear effects over long distances, leading to enhanced nonlinear effects, in particular, Kerr nonlinearity, SHG, THG, and FWM [147,148,149,150]. Doped semiconductors such as transparent conducting oxides (TCOs), for example, aluminium-doped zinc oxide (AZO) [151], and ITO [152], exhibit a near-zero permittivity at near-infrared frequencies, with the additional advantages of being CMOS compatible and of providing tunable platforms, whose ENZ frequency can be adjusted by controlling the doping level.

## 4. Conclusions

In this review, first, the main aspects of the most common fundamental NLO effects are described, pointing out that various types of all-optical functionalities can be implemented by nonlinear integrated photonic devices, based mainly on second- and third-order phenomena. Then, since the field of nonlinear photonics has been the target of constant innovations based on the investigation of many optical materials, the nonlinear optical (NLO) properties of most important optical materials are described. Silicon and related materials, such as SiN, a-Si, and SiC; glasses, such as silica, high-index glass, and chalcogenide glasses; and III–V semiconductors, in particular, AlGaAs, lithium niobate (LN), and recently investigated materials such as tantalum pentoxide (Ta_2_O_5_) and vanadium dioxide (VO_2_) are discussed, pointing out their pros and cons. Last, 2DLMs and zero-index media are described, too. We note that encouraging prospectives are provided by 2DLMs, but considering the production speed, yield, and quality, at the moment, the preparation of 2D materials is not sufficient to meet the requirements of industry or commercialization. In addition, the relatively low physical and/or chemical stability of some 2D materials makes them unsuitable for storage and the application of long-term stable optoelectronic devices.

A very interesting material platform, for instance, is scandium-doped aluminum nitride (Al_1−x_Sc_x_N), which maintains the CMOS compatibility of aluminum nitride (AlN) and exhibits a noticeable χ^(2)^ enhancement; for the Al_0.64_Sc_0.36_N composition, the χ^(2)^ component d_33_ reaches a value of 62.3 ± 5.6 pm/V, which is 12 times stronger than that of the intrinsic AlN and twice as strong as that of lithium niobate [153]. Another class of recently investigated materials are the phase-change materials (PCMs). In principle, PCMs are very promising to enable dynamic modification to the physical properties of devices at the microscale and nanoscale [117,118,119].

For more information on photonic structures, their fabrication issues, and recent developments and new progress in nonlinear photonics devices and system integration, the interested readers are referred to a companion paper [154].

## Figures and Tables

**Table 1:** Nonlinear refractive indices n_2_, two-photon absorption coefficients $\beta_{TPA},$ and figures of merit (FOMs) of various materials.

Material	$n_{2}\;\left(10^{-20}\frac{m^{2}}{W}\right)$	$\beta_{TPA}\;\left(\frac{\mathrm{cm}}{\mathrm{GW}}\right)$	Wavelength (nm)	FOM = n_2_/*β_TPA_λ*
SiO_2_ [103]	3	Negligible	1053	-
Hydex [52]	13	Negligible	1560	-
Stoichiometric SiN [76]	28	Negligible	1550	-
Lithium niobate [108]	39	Negligible	1064	-
4H-SiC [86]	60	Negligible	1545	-
Ta_2_O_5_ [91]	72	Negligible	800	-
Non-stoichiometric SiN [76]	140	Negligible	1550	-
Chalcogenides [106]	370	Negligible	1550	-
Crystalline Si [73]	400	0.8	1540	0.32
a-SiC [87]	480	Negligible	1550	-
Deuterated silicon-rich nitride [81]	980	Negligible	1550	-
GaP [99]	1100	Negligible	1550	-
AlGaAs [94]	1500	0.05	1550	19
Graphene/Si hybrid waveguide [98]	2000	0.5	1548	2.6
a-Si:H [82]	4200	4.1	1550	0.7
GaAs/AlAs superlattice waveguides [97]	5500	4	1505	0.9

## Data Availability

No new data were created.
